# Testing Assumptions of the Physical Activity Adoption and Maintenance Model: A Longitudinal Perspective of the Relationships Between Intentions and Habits on Exercise Adherence

**DOI:** 10.1177/00315125231188240

**Published:** 2023-07-09

**Authors:** Filipe Rodrigues, Diogo Teixeira

**Affiliations:** 1 ESECS, Polytechnique of Leiria, Leiria, Portugal; 2 Life Quality Research Center (CIEQV), Rio Maior, Portugal; 3 70887 Lusófona University of Humanities and Technology (ULHT), Lisbon, Portugal; 4 Research Center in Sport, Physical Education, and Exercise and Health (CIDEFES), Lisbon, Portugal

**Keywords:** PAAM, intention, habit, exercise

## Abstract

In this study, we aimed to examine empirically the Physical Activity Adoption and Maintenance model (PAAM). We collected data on these variables at baseline (T0) and 6-months later (T1). We recruited 119 participants (42 male, 77 female) aged 18–81 years old (*M*_age_ = 44.89, *SD* = 12.95). who reported, at baseline, that they exercised an average of 3.76 days per week (*SD* = 1.33) in training periods lasting 15–60 minutes (*M* = 38.69; *SD* = 23.28). We conducted hierarchical multiple regression analysis to test the association between each determinant (intentions, habits, and frequency) and future exercise adherence. We tested four models by entering blocks of predictors according to PAAM assumptions. The variance change (*R*^2^) between the first and fourth models (Δ*R*^2^ = .391) was statistically significant, showing that the fourth model accounted for 51.2% of variance for future exercise adherence, F (6, 112) = 21.631, *p* < .001, *R*^2^ = .73, adjusted *R*^2^ = .512. Exercise intention at T1 maintained its significant association (*p* = .021) with exercise frequency at T1 in all tested models. Exercise frequency at T0 was the most significant predictor (*p* < .01) of future exercise adherence, with past experience the second most significant predictor (*p* = .013). Interestingly, exercise habits at T1 and T0 did not predict exercise frequency at T1 in the fourth model. Among the variables we studied, having constantly high exercise intentions and high regular exercise frequency are significantly associated with maintaining or increasing regular future exercise behavior.

## Introduction

It is well-known in the realm of behavior research that most people fail to engage in sufficient physical activity to obtain the associated health benefits of avoiding chronic diseases, increasing longevity and functional autonomy, assisting their weight management, and improving their overall well-being (Warburton & Bredin, 2017). Several dominant social-cognitive and motivational theories have been adopted to explore the reasons and motivations underlying this insufficient physical exercise (Biddle et al., 2022), including self-determination theory (Ryan & Deci, 2017), the theory of planned behavior (Ajzen, 1991), and/or self-efficacy theory (Bandura, 1994). Investigators have conducted empirical studies (Brickell et al., 2006; Rodrigues et al., 2020; Tikac et al., 2022) based on these theories with promising results in the exercise context, explaining that autonomous motivation, perceived benefits, social support, and intentions are all significant predictors of exercise adherence. However, systematic research has also shown that the variance of behavior explained by these determinants is low (Rhodes et al., 2022); for example, 46% of those who intend to be physically active fail to translate their intentions into behavior (Rhodes & Bruijn, 2013).

Considering the intention-behavior gap (Rhodes et al., 2022), while exercise initiation is difficult, those individuals that are currently active tend to display their intentions to maintain the behavior for the medium-term future (Rodrigues et al., 2022). However, intentions may have less weight in explaining future exercise adherence once habit formation occurs. Possibly, individuals with less exercise experience have greater intentions to be physically active than more experienced exercisers who perceive the behavior as habitual (Gardner, 2015; Strobach et al., 2020). Empirical evidence shows that exercise experience has correlated with habit formation but not intentions (Tappe & Glanz, 2013). Exercise maintenance needs further study of the underpinnings of intentions and habits that lead individuals to maintain physical activity. Results of such research, based on dual process theory, may have practical implications in exercise science and physical activity promotion.

### The Exercise and Habit-Intention Relationship

Dual-process theories in the exercise context have been adapted from the other psychological literature to describe how individuals think and behave (Rhodes & Sui, 2021). These dual-process models describe how implicit and explicit processes continuously work together through mental assessments of the possibility of engaging (or not) in the proposed behavior. Implicit processes are intuitive, efficient, and based on pattern recognition and non-reflective thinking. A given behavior happens automatically, intuitively, and with little mental and/or physical effort. Explicit processes are reflective, require reason, and are based on cue assessment that requires conscious and logical thinking and more mental effort when compared to implicit processes (Strobach et al., 2020).

According to Gardner (2015), habit can be defined as generated impulses or behavioral patterns that can be learned through context dependent repetition. Thus, repeated performance in a constant setting reinforces cue-behavior associations such that just facing the context again is sufficient to automatically illicit a response to renew habitual behavior. For habit formation to occur, a behavior should be repeated frequently, in stable and consistent conditions, and there should be a steady decrease of the need for conscious-decision making (Gardner et al., 2020). On the other hand, intention is understood as an individual prompt to perform the behavior in the short- and medium-term time frame (Ajzen, 1991). A directed thought to perform a determined action implies the need for reflective thinking. Hence, for intention to be effective in stimulating a given behavior, rational processes are needed for changing other habitual behaviors or adopting new ones (Webb & Sheeran, 2006).

In the exercise context, intentions and habit seem to operate together, with different degrees (Bruijn & Rhodes, 2011). Theoretically, novice exercisers should display high intentions to be physically active, based on different logical thought and motivations (e.g., to achieve aesthetics, health, social recognition, physical fitness, etc.). This explicit intention process, while predominant, motivates novice exercisers to continue to exercise (Rodrigues et al., 2022). For these exercisers, habit should be close to zero, as the behavior has only been adopted recently. But if exercisers maintain their behavior adherence, then a decrease of this explicit process should be replaced by an increase in the implicit process of engaging more automatically in the actual behavior (Bruijn & Rhodes, 2011; Rebar et al., 2014; Rhodes & Sui, 2021). While this seems theoretically plausible, however, exercise is a complex behavior that requires preparatory behavior (e.g., organizing gym bag, going to the fitness center) and advanced thought (e.g., planning training days, following training regime), requiring a prolonged period of mental effort (Cheval et al., 2018). Thus, intentions might be high, even in experienced or well-practiced exercisers whose behavior is driven by motivations (e.g., be physically active for health-related reasons); while habit formation should increase as the behavior tends to be elicited by contextual cues (Gardner, 2015), this process can take longer and require ongoing self-regulatory skills and decision making for complex behavior like exercise (Hagger, 2019), making it more difficult to assume that exercise behavior has become non-conscious and automatic for experienced exercisers.

### Current Study

Recent studies have investigated the implications of dual-processes theories on the adoption and maintenance of exercise. The Affective-Reflective Theory (ART; Brand & Ekkekakis, 2018), the Affect and Health Behavior Framework (AHBF; Stevens et al., 2020), the Theory of Effort Minimization in Physical Activity (TEMPA; Cheval & Boisgontier, 2021), and the Physical Activity Adoption and Maintenance model (PAAM; Strobach et al., 2020) are examples of modern dual-process theories that integrate implicit and explicit processes towards physical activity and exercise behavior. Most of these theories consider affect to be an implicit component of the dual-process (i.e., ART, AHBF, TEMPA). The PAAM model also assumes that habit is an implicit exercise determinant that is complemented by intentions as an explicit determinant. PAAM developers, Strobach et al. (2020), assumed that implicit processes (e.g., habit, affect) are default responses on which explicit processes are based and developed (e.g., intention, self-regulatory trait, executive functions), similarly to the assumptions of the ART model (Brand & Ekkekakis, 2018). Depending on their intensity, these implicit processes can impact the explicit process of behavior engagement (Strack & Deutsch, 2004). In this context, implicit and explicit processes can be either concordant (e.g., the impulse to enjoy a fitness group class and the intention to increase physical activity levels) or conflicting (e.g., the impulse to rest on the couch after a day at work while the intention is to be more physically active). If self-regulation skills are available, then implicit processes may influence but not override explicit processing in determining exercise. That is, if an individual is intention-motivated with sufficient self-regulation capability, exercise will be guided most by this explicit process, but if there is low availability of explicit self-regulatory processing, the implicit process will dominate the behavior (Strack & Deutsch, 2004). In experienced exercisers, we might assume that self-regulatory ability is available since the individual tends to intentionally organize preparatory behaviors that lead to exercise frequency. Nonetheless, as experience increases, habit formation should increase and tend to regulate behavior by implicit processes, meaning that intentional effort should start to decrease as a means of minimizing mental effort (Cheval & Boisgontier, 2021),

Even in the context of dual-process theory, exercise is unlikely to become an exclusively non-reflective habitual behavior (Hagger, 2019); it will always require some degree of planning and explicit control. Thus, complex behaviors like exercise are likely to remain explicitly regulated, and habit may only increase to some degree over time, if the behavior is repeated in stable and consistent circumstances that are concordant with explicit eliciting determinants (Gardner, 2015; Strobach et al., 2020). The PAAM model also includes a moderating role of affect on the implicit side of the model, with continued executive functions and trait self-regulation on the explicit side. These moderators complement the prediction of future physical activity as they have some power in explaining how intentions and habits operate together towards the behavior as the main contributors to future exercise behavior.

While the PAAM model exemplifies this dual process theory in exercise adherence, empirical research to support it has been limited and has been based on only its explicit determinants (e.g., Finne et al., 2022; Rodrigues et al., 2022) or its implicit determinants (e.g., Faria et al., 2022; Teixeira et al., 2022). Additionally, most studies have been cross-sectional, limiting the interpretation of causal relationships between underlying variables, or have been weakened methodologically by a reliance on self-reported measures of exercise behavior (e.g., Rodrigues et al., 2021; Teixeira et al., 2022). As stated by Rhodes and Sui (2021), studies on exercise maintenance should be tested through longitudinal assessment of behavior change over time.

Should intentions decrease or be maintained, and should habit increase in individuals who have sustained exercise over time? There has been some evidence that intentions should decrease, and habits should increase across time as these theories would predict (Gardner, 2015; Strobach et al., 2020). However, there has been other evidence that intention persists for exercise action, this behavior is complex and needs continuous decision-making thinking (Hagger, 2019). We suspect that intentions and habits are both high in experienced exercisers and that both implicit and explicit dynamics explain exercise frequency over the long-term. In the present study, we aimed to examine the associations between exercise, intentions, and habits, using, as a reference, the PAAM model in a longitudinal perspective. We hypothesized that both habit and intentions at baseline would be associated with exercise frequency at baseline but not with exercise experience. We also speculated that intentions and habits at baseline would be associated with intentions and habits after 6-months of regular exercise practice. Following the PAAM model assumptions and exercise experience, we expected that future exercise adherence would be more associated with habits than intentions at baseline and after 6-months of exercise practice.

## Method

### Participants

We used an *a-priori* sample size calculator for hierarchical multiple regression analysis (Soper, 2023) to calculate the minimum required sample size for this study for sufficient statistical power. We assumed an anticipated effect size of .15 (medium effect), desired statistical power of .8; two predictors in set A and six in set B, and a statistical probability level of .05. The calculation suggested a minimum of approximately 86 participants.

Our recruited sample consisted of 119 participants (42 male, 77 female) aged 18–81 years old (*M*_age_ = 44.89, *SD* = 12.95). Participants all reported at baseline that they were exercising on average 3.76 days per week (*SD* = 1.33) with training periods lasting between 15 and 60 minutes (*M* = 38.69; *SD* = 23.28). Participants were actively engaged in fitness group classes (*M*_minutes_ = 33.25; *SD* = 25.28) or aerobic/resistance training (*M*_minutes_ = 44.12; *SD* = 19.75). Concerning their body mass index (BMI), participants self-reported their height and weight to be normal (62.2%), overweight (27.7%), or obese (10.1%). Participants met the following inclusion criteria: (a) aged 18 years or older; (b) provided informed consent to participate; and (c) were a member of an active fitness center. Active fitness center members were defined as individuals who maintained an active monthly subscription to a fitness center or gym and engaged in exercise at least twice per week at the facility. They were actively engaged in fitness-related activities and exercise programs provided by the facility.

We obtained approval for this research protocol from our university IP Leiria (CE/IPLEIRIA/35/2021) institutional ethics committee prior to conducting the study, and we obtained written informed consent from all participants. Following ethical institutional board approval, we contacted several fitness facilities (*n* = 4) and explained our research objectives and data collection procedures individually to the managers. After approval, potential participants were contacted at the reception desk prior to the training session and were asked to participate voluntarily in this study. Objectives for this study were explained to all participants, and signed informed consent was obtained individually.

### Assessment Instruments

Participants completed several paper-and-pencil self-administered questionnaires whose total administration time required less than 10 minutes. We measured participants’ *intentions* towards exercise with the validated Portuguese scale (Rodrigues et al., 2020) for this purpose, grounded on the theory of planned behavior. Three items to evaluate intention to continue exercising (e.g., “*I will continue to exercise in the next 6 months as I currently do*”) were created and responded using a seven-point scale anchored from 1 (absolutely not) to 7 (absolutely yes). The employed scale has demonstrated robust levels of both validity (Comparative Fit Index [CFI] = .93; Tucker-Lewis Index [TLI] = .91; Standard Root Mean Residual [SRMR] = .05; Root Mean Square Error of Approximation [RMSEA] = .05) and reliability (alpha coefficient = .89), as also evidenced by acceptable scores in prior research (Rodrigues et al., 2020).

We used the Self-Report Behavioral Automaticity Index – Portuguese version (Rodrigues et al., 2021) to measure the degree of the participants’ exercise *habits*. A seven-point scale ranging from 1 (strongly disagree) to 7 (strongly agree) was used to respond to four items (e.g., “*Exercising is something I do automatically*”). The employed scale has demonstrated robust levels of both validity (CFI = .97; TLI = .92; SRMR = .03; RMSEA = .05) and reliability (alpha coefficient = .93), as also evidenced by acceptable scores in prior research (Rodrigues et al., 2021).

Exercise *frequency* at baseline was measured using a single-item question (“*During the last 7 days, on how many days did you exercised?*”). After 6-months of exercise, we calculated weekly mean scores to compare their exercise adherence 6 months after baseline, based on electronic log attendance. Previous literature has used these methods on measuring exercise frequency over a longitudinal perspective (Rodrigues et al., 2022).

For measuring the participants’ exercise *experience*, we explored computer records of participants’ self-reported data. For this study, exercise experience was considered as the registration date, and data was coded in months. This variable was created based on previous assumptions (Rodrigues et al., 2022).

### Statistical Analysis

All analyses were conducted using SPSS Version 26.0 software (IBM Corp., Armonk, NY). We used the expectation-maximization approach to handle possible missing data completely at random. We reported descriptive statistics in means (and standard deviations) and in frequency percentages, and to determine the statistical significance of deviation from a normal distribution, we estimated the skewness and kurtosis of the distributions and divided them by their corresponding standard error to get the z score. A Z-score below |1.96| suggested a normal distribution (Cohen, 1988). Bivariate correlations were conducted on variables of interest, and partial correlations were performed controlling for sex. We set the significance level t at *p* ≤ .05 to reject the null hypothesis. Alphas for internal consistency were calculated considering acceptable coefficients as ≥ .70 (Cronbach, 1951).

We conducted hierarchical multiple regression analyses to test the proposed associations. Before performing regression analysis, we analyzed tolerance test and Variance Inflation Factor (VIF) scores to test for possible multicollinearity issues (Cohen, 1988). The tolerance of independent variables should be greater than 0.1 for there to be no multicollinearity. The Durbin Watson statistic test for autocorrelation was also calculated, assuming an acceptable range of 1.50–2.50 (Durbin & Watson, 1950). Six-month exercise adherence was imputed as the dependent variable. We used the stepwise regression procedure, as we intended to add variables following the PAAM model assumptions as detailed in the Introduction to this paper. We inserted the participants’ self-reported intentions and habit at T1 in block 1, and we inserted self-reported exercise frequency at T1 in block 2. In block 3, we inserted their intentions and habits at T0, and in block 4, we inserted their exercise experience. We compared regression models using *R*^2^ and we analyzed changes in the adjusted *R*^2^ using the significance level of ≤ .05 to reject the null hypothesis.

We then used paired sample t-tests to compare frequency, intentions, and habits between T0 and T1. We calculated effect sizes based on Cohen’s *d,* setting thresholds of .2, .5, and .8 as ‘small,’ ‘medium,’’ or ‘large’ effects (Cohen, 1988). Again, a significance level of *p* ≤ .05 was set to reject the null hypothesis.

## Results

The participants’ descriptive statistics, bivariate correlations for variables of interest, and internal consistency coefficients are shown in Table 1. Across all participants, mean scores for exercise frequency at baseline were greater than mean scores of exercise frequency at T1. Exercise intentions decreased from T0 to T1, but habits increased over the course of these 6-months. Data distributions for skewness (except for intention at T1) and kurtosis values were below the cutoff (except for intention at T0 that was over but close to cutoff) indicating normal distributions. Several significant bivariate correlations emerged as expected, namely: (a) exercise frequency at baseline, exercise intentions at T0 and T1 (*p* = .002 and *p* = .03, respectively), and exercise habits at T0 and T1 (*p* = .002 and *p* = .004, respectively) were positively and significantly correlated with each other; (b) both measurement points of intentions were positively and significantly correlated (*p* = .002) with each other; (c) both measurement points of habits were positively and significantly correlated (*p* < .001) with each other; and (d) frequency at T1 was positively associated with intentions at T1 (*p* = .001) and habits at T1 (*p* = .001). Partial correlations displayed similar results compared to those from the bivariate correlations, since significance levels were maintained in all tested associations. Alpha coefficients were all above .70, showing acceptable internal consistency.

**Table 1.** Participants’ Descriptive Statistics and Internal Consistency Coefficients, and Bivariate and Partial Correlations of Variables of Interest.

Variables	*M*	*SD*	S	K	1	2	3	4	5	6	7	α
1. Exercise experience	11.74	8.89	.70	−.61	1	.02	.08	.08	.08	.08	.33*	—
2. Intention (T0)	6.36	.89	−1.7	2.25	.01	1	.12	.29*	.29**	.16*	.15*	.90
3. Habit (T0)	4.41	1.50	−.20	−.80	.08	.11	1	.22*	.22*	.41**	.26**	.85
4. Frequency (T0)	3.76	1.33	.24	−.40	.08	.28^**^	.22*	1	.20**	.26*	.65**	-
6. Intention (T1)	6.23	1.24	−2.03	1.61	.09	.28^**^	.22**	.20^**^	1	.38**	.29**	.97
7. Habit (T1)	4.74	1.41	−.32	−.64	.04	.15^*^	.51^**^	.26^*^	.38^**^	1	.28**	.87
8. Frequency (T1)	3.61	1.53	1.38	1.29	.34**	.15^**^	.26**	.65^**^	.30^**^	.29^**^	1	—

*Note*: *M* = Mean; *SD* = Standard-Deviation; S = Skewness; K = Kurtosis; α = Internal consistency coefficient; below diagonal line = bivariate correlations; above diagonal line = partial correlations controlling for sex; *p < .01; **p < .01.

Hierarchical multiple regression results are presented in Table 2. The tolerance values ranged from .65 to .92. In addition, the VIF values ranged from 1.09 to 1.54. Therefore, there was no multicollinearity issues in this analysis. The Durbin–Watson test indicated a score of 1.93, indicating score close to zero autocorrelation. We checked the significance (*p*-value) of each of the models to examine whether the model was significantly different from a null hypothesis. We checked the *R*^2^ value to see how much of the variance of future exercise adherence was explained by the model. To identify which variable contributed most to the model, we checked the standardized coefficients and significance of the independent variables. In hierarchical multiple regression analysis, we compared the models as variables were added (changes in *R*^2^).

**Table 2.** Hierarchical Multiple Regression Analysis.

	β	t	df	R	*R* ^2^	Δ *R*^2^	df	*F*
Block 1			2, 116	.35	.11	.12	2, 116	.001
Intention T1	.22*	2.35						
Habit T1	.20*	2.07						
Block 2			3, 115	.67	.44	.33	1, 115	.000
Intention T1	.15*	2.01						
Habit T1	.06	.84						
Frequency T0	.60**	8.29						
Block 3			5, 113	.68	.44	.01	2, 113	.201
Intention T1	.17*	2.26						
Habit T1	.02	.24						
Frequency T0	.61**	8.29						
Intention T0	−.11	−1.43						
Habit T0	.09	1.12						
Block 4			6, 112	.73	.51	.07	1, 112	.000
Intention T1	.15*	2.11						
Habit T1	.03	.51						
Frequency T0	.60**	8.59						
Intention T0	−.10	−1.39						
Habit T0	.07	.91						
Exercise experience	.27**	4.16						

*Note*: β = standardized coefficients; t = *t* test; *R*^2^ = adjusted r square - explained variance; Δ = differences; F = changes in significance; *p < .01; **p < .01.

In the first model, the regression model was statistically significant and showed that participants’ exercise intentions and habits at T1 accounted for 11.0% of the variance in their later exercise adherence, F (2, 116) = 7.91, *p* < .001, *R*^2^ = .35, adjusted *R*^2^ = .11. The second model accounted for 44% of the variance on future exercise adherence, F (1, 115) = 68.69, *p* < .001, *R*^2^ = .67, adjusted *R*^2^ = .44, by considering frequency at T0. The third model accounted for 44% with no significant statistical increase and the last model accounted for 51% of variance in frequency at T1, F (1, 112) = 17.317, *p* < .001, *R*^2^ = .73, adjusted *R*^2^ = .51. The change of *R*^2^ between the first and fourth models (Δ*R*^2^ = .40) was statistically significant. These results indicate that the last model was the most parsimonious compared to the previous models.

Exercise intention at T1 maintained its significant association (*p* < .05) with exercise frequency at T1 in all tested models. Frequency at T0 was the most significant predictor (*p* < .01) of later exercise adherence, with exercise experience the second most significant predictor (*p* < .05). Interestingly, habit at T1 and T0 did not predict exercise frequency at T1 in the fourth model.

The results of the paired sample T-tests are presented in Table 3. There were no significant differences in exercise frequency and intentions between baseline and T1 (*p* > .05). There were differences in habits having a significant increase (*p* < .05), showing a medium effect sizes *d* = .22 (for details see Table 3).

**Table 3.** Paired Sample t-tests Analyses.

	Mean (SD)	Mean (SD)	Change	*t*	*p*	*d*
	Baseline (T0)	T1				
Frequency	3.76 (1.33)	3.61 (1.53)	.15 (.20)	1.35	.178	—
Intention	6.36 (.89)	6.23 (1.25)	.13 (.36)	1.00	.319	—
Habit	4.41 (1.50)	4.74 (1.41)	.33 (0.9)	−2.49	<.05	.22

*Note*: *t* = paired sample *t* test; *d* = Cohen’s d.

## Discussion

In the present study, we aimed to test PAAM assumptions of a dual process involvement of exercise intention and exercise habit on exercise behavior over a longitudinal 6-month perspective. Our results partially supported our first hypothesis, since exercise *experience* was not associated with exercise frequency, intentions, and habits at baseline. On the other hand, exercise intentions and habits at baseline were significantly associated with exercise *frequency* (T0), with the correlation coefficient between intentions and exercise frequency stronger compared to the same correlation between habits and frequency. This finding offered support to some existing studies (Bruijn & Rhodes, 2011; Rodrigues et al., 2021), showing that, while individuals were in the maintenance phase of exercise behavior, high exercise intentions were still needed to motivate exercise practice. Remarkably, after 6-months of adherence to exercise, habits at baseline (T0) correlated to future exercise adherence at similar levels to the correlation between intentions at baseline (T0) and future exercise adherence. A similar trend emerged in the associations between habits and intentions at T1 (6 months later). This result corroborates with existing literature (Gardner, 2015; Strobach et al., 2020), suggesting that increased exercise repetition over time in in the context of stable and consistent conditions increased the likelihood that exercise would be perceived as more habitual, compared to intentional behavior.

These results support the PAAM model, by showing how explicit (intention) and implicit (habit) variables related to dual-process assumptions of their joint operation in explaining later exercise frequency. In the current study, the explained variance of later exercise adherence from the intention and habit model was 52%, showing some utility to this understanding of exercise behavior. The participants displayed consistent intentions (at both T0 and T1) towards exercise, and intention was significantly associated with later exercise adherence. This explicit determinant seems to predict exercise maintenance, suggesting that some cognitive function is still needed, even after 6 months of exercise practice, for the behavior to be elicited (Cheval et al., 2018). This continuing relevance of intention is supported by some prior studies (e.g., Finne et al., 2022; Rodrigues et al., 2022), emphasizing that, for the exercise behavior to be persistently performed, ongoing decision-making processes are needed, even for individuals with ample exercise experience. Exercise frequency at baseline also showed a positive and significant association with exercise adherence after 6-months. Rodrigues et al. (2022) described this significant association as a continued need for explicit decision-making for individuals to be physically active. While there could be a shift in motives and reasons across time (e.g., initiating exercise practice for extrinsic motives and then maintaining the behavior for the identified motivation), intentions retained significance in the prediction of future exercise adherence.

According to the PAAM model, both intentions and habits at baseline should be associated with intentions and habits after 6-months of regular exercise frequency among experienced exercisers. Our results supported these assumptions, as we found that the associations between T0 and T1 were significant for both intentions and habits. In addition, habit did increase across 6-months of exercise, supporting the view that habit formation derives from repeated behavior (Gardner, 2015). While intentions decreased, this decline was not significant, and intentions were still a predictor of later exercise adherence. Thus, as habit formation is facilitated by a number of processes, new knowledge of the relative contribution of intentions and habits in the process of exercise adherence over time enhances our understanding how to create efficient interventions for promoting physically active behaviors. Our findings revealed the importance of both explicit intention and implicit habit in the perpetuation of exercise over a 6-month period.

### Limitations and Directions for Future Research

While this study was longitudinal in nature, we used no intervention and cannot infer a causal relationship between our variables of interest and exercise adherence. Further experimental work could provide clear evidence on the differential timing influence of explicit intentional and automatic habit processes (Rebar et al., 2014). In addition, exercise intentions habits and frequency were self-reported in this study, meaning that participants may have over- or under-estimated the extent of their influence on exercise behavior (Rodrigues et al., 2022). Accelerometers and/or pedometer, as well as electroencephalogram are tools to be considered in future research on physical activity adoption and maintenance (Cheval et al., 2018). While we explored intentions, habits, and exercise behavior at two time points, forthcoming studies might further increase our understanding of the PAAM model by assessing affect and executive functions and trait self-regulation factors of this theory (Strobach et al., 2020) and might study exercise adherence over a still longer period. Anther limitation that could have influenced our results is sample size. While we conducted an a priori power analysis to estimate our required sample size, larger samples would enhance the generalizability of these data and permit analyses of moderating variables (e.g., sex, fitness activity, body mass index) that could further increase our understanding of the predictive power of intentions, habits, and exercise behavior across time. Last, we provided evidence of the applicability of the PAAM model, using data retrieved from exercisers with some experience. Future studies could compare these results to novice exercisers who only recently adopted exercise behavior (<1 month experience).

## Conclusions

In this study, exercise frequency at baseline was the most significant predictor of exercise frequency 6 months later, with exercise experience the second most significant predictor. Exercise intentions at baseline were significantly associated with later exercise in all tested models, but habits at both time points did not predict later exercise frequency in the fourth model. Having intentions and regular exercising have the potential to maintain or increase later regular exercise among experienced exercisers. However, habits significantly increased over a period of 6-months, suggesting that individuals were gradually experiencing exercise as a habitual behavior, that permits habit formation to later contribute to this behavior’s maintenance. Exercise promotion efforts that attend to both habit development and intention maintenance may enhance the effectiveness of exercise interventions directed at encouraging individuals to adopt and maintain exercise behavior over time.

It is becoming increasingly clear that dual-process assumptions best apply to promoting exercise. In addition to increasing or maintain intentions towards future exercise adherence, exercise interventions may also benefit from enhancing automatic processes, such as habits, to help compensate for times when physical activity intentions are weak. In the present study we provided empirical support for the dual process PAAM model and the need to use measures of intentions and habit to further our understanding of exercise adherence. Our findings indicate that exercise behavior has not only a cognitive and planned aspect but also a habitual component, with both important to exercise maintenance. Our results demonstrate that intentions retain predictive power on exercise adherence over the medium term. Strong habits may be a viable predictor for explaining variance in exercise behavior, but PAAM assumptions of a dual process seem to correctly emphasize the combined importance of both explicit intent and implicit habit in exercise maintenance.

## Data Availability

The data that support the findings of this study are available upon reasonable request from the corresponding author. The data are not publicly available due to privacy and ethical restrictions.
